# Editorial: Experts' opinion in public health policy

**DOI:** 10.3389/fpubh.2023.1166645

**Published:** 2023-03-21

**Authors:** Stefania Salmaso

**Affiliations:** Independent Researcher, Rome, Italy

**Keywords:** climate change, decarbonization, retirement, cannabis, real world data, inequalities

Public health has an important contribution to make in developing and implementing policies in a wide variety of fields. In recent years, our attention has been largely monopolized by the COVID-19 pandemic, which has been the topic of the majority of scientific journal submissions. In late 2022, we established a Research Topic entitled “*Expert's opinion*” to publish short articles on a wide variety of public health issues that are relevant for policy setting, but concern topics that are not often reported on in our journal. In some cases, we invited leading scientists in specific fields to submit articles. In others, the “*Expert's opinion*” has included spontaneous submissions on a topic important to public health policy. It is our hope that this collection is of general interest to our readers and provide the opportunity to learn about different fields. Below we illustrate the breadth of topics where public health has important policy relevance and where new methodologies are needed to ensure that policy is based on sound scientific evidence.

One of the articles examined the difficulties faced in establishing official regulations for medical cannabis (Fortin et al.) and the use of alternative sources of data for regulatory approval. The authors underline the need for a reconciliation of the traditional empirical use of cannabis with the evidence-based medicine and the importance of regulatory approval, but they also outline the difficulties in obtaining the required evidence. Historically, randomized clinical trials (RCT) are considered the gold standard. They are required to obtain regulatory approval of use of a medical substance and are important in defining indications for its use and establishing recommendations and policies in public health. However, with medical cannabis, obtaining meaningful data for such approval is complicated by the great variability of the product. This article makes a case for the use of real-world data on the medical use of cannabis in the approval process, given the potential benefit of cannabis for a wide variety of patients. Observational studies will also be essential to assess potential health risks posed by cannabis use in different settings.

Another paper in the collection also stresses the tension between RCTs and the use of other data collection methods to support policy and focuses on the reluctance of health institutions and academies to more widely participate in generating and supporting the use of real-world data (Corrao et al.). The use of real-world data is likely to gain further importance as medicine puts increasing emphasis on so-called “precision medicine” and “personalized healthcare strategies” targeting specific patient subgroups. The conventionally used RCTs are characterized by rigorous inclusion criteria, and although they can control for potentially confounding factors, they do not allow to study the effects of a drug on the variety of patients, who will be exposed to it in the real life, where the effects of treatment may be considerably different. This underlines the importance of involving institutions and academies in improving the quality of real-world data and promoting research on methodological approaches and the use of current data to complement RCT results.

The issue of climate change has important effects also on public health and the paper by Weinmayr and Forastiere underlines the relationship between air quality and public health. Air pollution is the fourth leading risk factor for premature deaths, not only from respiratory diseases, but also diabetes and dementia. Public health therefore is not only an important stakeholder (actually a core one) for the implementation of actions against climate change, but to further increase its impact, it needs to identify and employ innovative methods for estimating the health impact of climate policies. Systems thinking and co-production are the two concepts to address the challenges with a holistic approach and should become more commonly adopted in the public health community.

Because climate change is a challenge for all sectors of our society, it is time for the health service sector consider monitoring and reducing its carbon footprint, as addressed by Armocida et al. in a paper in our collection. Such efforts are in line with the commitment made by 50 countries during the COP26 to develop low-carbon health systems. The paper identifies the European Green Deal as the best opportunity to put in place actions to combat climate change. However, it underlines that most actions are oriented to adapting to climate change instead of reducing carbon-related emissions and points out the example of Italy, which has not even undersigned the pursuit of such goals., although the carbon footprint of its health system is estimated to be 4% of the national footprint. Public health policies should be developed and implemented to limit emissions by re-organization of health care, for example by using telemedicine to reduce patient travel and improving the digitalization and easy access of health records. The issue of reducing the health sector's carbon footprint is of wide interest beyond Italy, and additional studies are warranted to measure and provide evidence about the actual impact of actions undertaken (1).

During the recent pandemic, most people perceived health and economy as two conflicting priorities. However, in reality, the two are closely related, with the economy playing an important role in health and health being is a key determinant of the economy. As emphasized in the paper in this issue by Ardito and Costa, it is important to develop policies to prevent and compensate health inequalities and their economic consequences. An important example about the interplay between health policy and economics can be seen in the current debates over retirement age in many Western countries. The retirement age in these countries was set decades ago based on the life expectation at that time, and several countries have moved to change it as overall life expectancy has increased. However, there is an interaction between inequalities in health and longevity and social security rules, and the authors argue that these inequities should be considered when governments take actions to ensure the sustainability of pension systems. The authors argue for an increase in governmental flexibility around the age at which individuals can retire. Public health can play an important role in economic policy by developing the scientific evidence to disentangle the causal pathways linking low socioeconomic status to shorter life expectancy by identifying direct and reverse causative factors. Monitoring data on health inequality and using the findings on the differences in life expectancy between socioeconomic groups to guide the implementation of preventive and compensatory policies in each step of the life of each person are essential to reduce inequity and its economic impacts.

## Author contributions

The author confirms being the sole contributor of this work and has approved it for publication.
